# The green and blue crop water requirement WATNEEDS model and its global gridded outputs

**DOI:** 10.1038/s41597-020-00612-0

**Published:** 2020-08-18

**Authors:** Davide Danilo Chiarelli, Corrado Passera, Lorenzo Rosa, Kyle Frankel Davis, Paolo D’Odorico, Maria Cristina Rulli

**Affiliations:** 1 Department of Civil and Environmental Engineering, Politecnico di Milano, 20133 Milan, Italy; 2 Department of Environmental Science, Policy, and Management, University of California, Berkeley, 94720 Berkeley USA; 3 Department of Geography and Spatial Sciences, University of Delaware, 19716 Newark, USA; 4 Department of Plant and Soil Sciences, University of Delaware, 19716 Newark, USA

**Keywords:** Hydrology, Agriculture, Water resources

## Abstract

Accurately assessing green and blue water requirements from croplands is fundamental to promote sustainable water management. In the last decade, global hydrological models have provided important insights into global patterns of water requirements for crop production. As important as these models are, they do not provide monthly crop-specific and year-specific data of green and blue water requirements. Gridded crop-specific products are therefore needed to better understand the spatial and temporal evolution of water demand. Here, we present a global gridded database of monthly crop-specific green (rain-fed) and blue (irrigated) water requirements for 23 main crops and 3 crop groups obtained using our WATNEEDS model. For the time periods in which our dataset matched, these estimates are validated against existing global products and satellite based datasets of evapotranspiration. The data are publicly available and can be used by practitioners in the water-energy-food nexus to assess the water sustainability of our food and energy systems at multiple spatial (local to global) and temporal (seasonal to multi-year) scales.

## Abstract (summary)

**Table:** 

Measurement(s)	crop-specific green water requirements • crop-specific blue water requirements
Technology Type(s)	computational modeling technique
Factor Type(s)	crop type • year of data collection • monthly measurement
Sample Characteristic - Environment	area of cropland
Sample Characteristic - Location	Earth (planet)

Machine-accessible metadata file describing the reported data: 10.6084/m9.figshare.12706523

## Background & Summary

Water plays a central role in supporting agriculture, with food production responsible for ~90% of humanity’s consumptive water footprint^1,2^. Accurately assessing demands for freshwater resources – both in terms of timing and magnitude – is critical for understanding the water sustainability of agriculture. A better understanding of agricultural water needs could be used to identify those places where water demand and its variability could potentially compromise the reliability of food production, and for formulating solutions to promote sustainable water management. Some studies have helped to expand our knowledge of crop water use by adopting the water footprint approach^3,4^ that partitions crop water requirements (i.e., the volume of water needed to support crop’s evapotranspiration during its growing period without experiencing water stress) between blue water and green water (i.e., water from water bodies or aquifers and soil moisture, respectively). Some studies have provided estimates of annual trends in water demand from croplands^5,6^ while others have progressed to finer temporal (i.e., monthly) resolutions that consider multiple individual crops^4,7^. The latter crop-specific studies have been typically centred on the year 2000, offering an important snapshot of the global distribution of water demand and insight into the importance of certain crops in driving water demands in different regions. More broadly, all of these products have helped to advance the science of food security and water sustainability and to identify those places where chronic or seasonal water stress is occurring because of agricultural water demand.

Unlike the spatial distribution of crop water demand, the time component of freshwater demand is less well understood. To date, most temporal studies of water use in agriculture have concentrated on national, regional, or basin scales (e.g., refs. ^8–10^) or examined the agricultural sector as whole (e.g., refs. ^9,11^) and have rarely captured the intra-national and intra-annual dynamics of water demand and availability. In addition, previous studies examining long-term temporal trends in crop water use have relied primarily on national, temporally averaged crop water requirement values (mm H_2_O yr^−1^; see e.g., ref. ^12^). These values are then typically multiplied by a crop’s annual harvested area in a particular country to determine the total water demand for that crop in that year. While such analyses provide important information related to broad trends of water use for crop production, such an approach does not permit temporal examinations that are spatially explicit and intra-annually disaggregated or that account for different crop growing periods. As such, there is a persisting need for datasets that not only incorporate the advances of previous work (i.e., spatially explicit, crop-specific) but that also expand upon these strengths by adding the dimension of time.

Here we address this important gap by presenting our WATNEEDS model. The model assesses the vertical component of the soil water balance and introduces a spatially distributed crop specific monthly analysis of green and blue water requirement and use for available climatic data. Specifically, evapotranspiration is computed using the FAO Penmann Monteith method^13^, overland flow is modelled as soil water saturation excess mechanism, and percolation is a linear function of the maximum infiltration rate and soil moisture, as in Hoogeven *et al*.^14^. As input data for WATNEEDS model, we use MIRCA crop distribution data^15^ and crop parameters and growing stage data taken from Allen *et al*.^13^, following Siebert and Döll^7^. We provide WATNEEDS model outputs for the 1998–2002 average (i.e., circa year 2000) and for the year 2016. Moreover, we provide monthly crop-specific gridded maps for 5 main crops.

Specifically, we begin by describing the biophysical input datasets required for the calculation of crop water requirements including spatially and temporally distributed information on climate, soil, and crop characteristics. We then describe how the model (WATNEEDS) solves a daily vertical soil water balance to generate estimates of green and blue crop water requirements. Using the model WATNEEDS, we present a global gridded (5 arcminute) dataset of monthly green and blue crop water requirements for 5 major crops and annual green and blue crop water requirements for 23 crops and 3 crop groups that encompass the vast majority of global water use for food production for the average year 2000 (i.e. averaged among 1998–2002 yearly simulations) in agreement with previous datasets and the most recent available year 2016. Moreover, where temporal and spatial overlap permits, the model outputs generated by this study are also compared to other existing global model outputs and satellite-derived products Ultimately, this model will be used to develop a continuous global time series for better understanding the spatial and temporal heterogeneity and variability of freshwater demand for food production as well as how water demand has evolved through time relative to water availability. Such historical examinations can provide insights for more accurate predictions of future water demand and availability.

## Methods

This section provides a detailed description of the input data sources, the model components used for calculating crop water requirements, and the resultant time series of global gridded monthly crop water requirement maps.

The crop water requirement (mm yr^-1^) is the volume of water required to compensate for a crop’s evapotranspiration losses and to prevent crop water stress. This crop water requirement can be divided into two components: the green crop water requirement (met by available precipitation) and the blue crop water requirement (met by irrigation). The crop water requirement is fully satisfied only when there is enough water for the plant to take up during its growth (i.e. enough precipitation or irrigation) without undergoing water stress. In regions of the world where crop water demand cannot be met by rainwater, only part of the crop water requirement is satisfied by green water (i.e. actual evapotranspiration). Irrigation can be used to supplement the crop’s water needs, thereby allowing crops to evapotranspire at the potential rate. For the years around 2000 (i.e., looking at average results for 1998–2002) and the year 2016, we calculated yearly blue and green crop water requirements for 23 major crops – barley, cassava, citrus, cocoa, coffee, cotton, date palm, grapes/vine, groundnuts/peanuts, maize, millet, oil palm, potatoes, pulses, rape seed/canola, rice, rye, sorghum, soybeans, sugar beet, sugar cane, sunflower, and wheat – that currently account for 76% of global crop production and 95% of global harvested area^16^ and 3 crop groups (fodder grasses, others annual crops, and others perennial crops). Specifically, we estimated actual green water use in rainfed areas and green and blue water use in irrigated areas. We also assessed monthly green and blue water requirements for five major crops – wheat, maize, rice, sugarcane and soybean – that currently account for almost half of global crop production. Land use, soil characteristics, crop calendars and crop growing stages are kept constant in all years using values available for the year 2000.

### Data sources

Monthly data on potential reference evapotranspiration (*ET*_*o*_) came from the University of East Anglia’s Climate Research Unit Time Series version 4.01 dataset (CRU TS v. 4.01; 0.5° × 0.5° resolution)^17^ and was calculated using the Penmann-Monteith equation, following Allen *et al*.^13^. Daily precipitation data between the latitudes 50° N and 50° S came from the Climate Hazards Group InfraRed Precipitation with Station version 2.0 dataset (CHIRPS; 0.05° × 0.05° resolution)^18,19^ while precipitation data for the remaining latitudes was taken from the National Oceanic and Atmospheric Administration’s Climate Prediction Center Global Unified Gauge-Based Analysis of Daily Precipitation dataset (CPC; 0.5° × 0.5° resolution)^20^. Soil information – maximum soil moisture storage capacity and maximum infiltration rate – were from Bajties *et al*.^21^ (0.08333° × 0.08333° resolution). Crop coefficients (*k*_*c*_) and growing stages came from Allen *et al*.^13^ Growing stages – originally reported as a percentage of the growing period of a crop – were then scaled to the planting and harvesting dates reported for the 402 regions and sub-regions included in the MIRCA2000 dataset^15^. Crop-specific rooting depths for irrigated and rainfed crops and critical depletion factors came from Allen *et al*.^13^. All gridded datasets were resampled to a 5 arcminute (0.08333°) spatial resolution.

### Multiple growing seasons

For a number of regions included within the MIRCA2000 dataset^15^, more than one growing period is reported for certain crops. This is true for irrigated rice and wheat. For cases where more than two growing periods were reported, we averaged the growing periods with the harvested area reported by Portmann *et al*.^15^. In a limited number of cases, the harvested areas were reported as equal across all growing periods for a particular crop and region. In these instances, selection of the two dominant growing periods was complemented using the growing periods of Mekonnen and Hoekstra^4^, based on USDA^22^ and FAO^23^ information.

### Atmospheric demand on crops

Evapotranspiration represents the rate of water flow to the atmosphere as water vapor. Potential evapotranspiration corresponds to the crop water requirement of plants (CWR) in the absence of water-stress; it can be reached when plants can take up from the soil the amount of water they need. This water comes from precipitation (green water - GW) and, in the case of deficiency, it is supplemented by irrigation (blue water - BW).

Potential evapotranspiration $E{T}_{i,t}\left(\frac{mm}{day}\right)$ can be assessed as

1 $$E{T}_{i,t}={k}_{c,i,t}\times E{T}_{o,t}$$

where *k*_*c,i,t*_ (−) is the crop coefficient of crop *i*, corresponding to the growing stage in which day *t* occurs; crop coefficients are taken from Allen *et al*.^13^. *ETo* is the reference evapotranspiration^17^.

The daily actual evapotranspiration (*ET*_*a,i,t*_) $\left(\frac{mm}{day}\right)$ of crop *i* on day *t* is then calculated as:

2 $$E{T}_{a,i,t}={k}_{s,i,t}\times E{T}_{i,t}$$

where *k*_*s,i,t*_ (−) is the water stress coefficient calculated as a function of the soil water content in the root zone (*S*_*i,t*_) and the maximum and actual water content in the root zone, as in Allen *et al*.^13^. For crop *i* on day *t* under water stressed conditions (i.e., when only precipitation is provided), *k*_*s,i,t*_ was evaluated as:

3 $${k}_{s,i,t}=\{\begin{array}{cc}{\frac{{S}_{i,t}}{RAW}}_{i} & \,if\,{S}_{i,t} < RA{W}_{i}\\ 1 & \,if\,{S}_{i,t}\ge RA{W}_{i}\end{array}$$

where *S*_*i,t*_ (mm) is the depth-average soil moisture and *RAW*_*i*_ (mm) is the readily available water. *RAW* is calculated as:

4 $$RA{W}_{i}={p}_{i}\times TA{W}_{i}={p}_{i}\times ({\theta }_{fc}-{\theta }_{wp})\times {z}_{r,i}$$

where *TAW*_*i*_ (mm) is the total available water (i.e., the amount of water that a crop can uptake from the rooting zone), *p*_*i*_ (−) is the critical depletion factor (i.e., the fraction of *TAW*_*i*_ that a crop can uptake from the rooting zone without experiencing crop water stress), $${\theta }_{fc}-{\theta }_{wp}$$$$\left(\frac{mm}{m}\right)$$ is the maximum soil moisture storage capacity dependent on soil texture (i.e., the difference between the water content at field capacity and the water content at the wilting point)^14^, and *z*_*r*_ (m) is the crop rooting depth^7^. For conditions of no water stress (where supplementary irrigation is available), *k*_*s,i,t*_ was assumed to be equal to 1 (see ref. ^13^).

### Vertical soil water balance

For a given crop and grid cell, soil moisture (*S*_*i,t*_) was calculated by solving a daily soil water balance:

5 $${S}_{i,t}={S}_{i,t-1}+{\Delta }t\times ({P}_{eff}-E{T}_{a,i,t}-{D}_{i,t}-{R}_{i,t})$$

where *S*_*i,t-1*_ (mm) is the soil moisture of the previous time step, *Δt* is equal to one day, *P*_*eff*_ $\left(\frac{mm}{day}\right)$ is the effective precipitation – where we assume that 5% of precipitation is partitioned to surface runoff following Hoogeveen *et al*.^14^, *I*_*i,t*_ $\left(\frac{mm}{day}\right)$ is the additional irrigation water (used only in the case of irrigated crops), and *R*_*i,t*_ $\left(\frac{mm}{day}\right)$ is the sub-surface runoff. *D*_*i,t*_ $\left(\frac{mm}{day}\right)$ is deep percolation below the root zone (which occurs when soil moisture exceeds field capacity (i.e., the volume of water able to be retained in the soil)) and was calculated as:

6 $${D}_{i,t}=\{\begin{array}{cc}{F}_{max}\times \frac{{S}_{i,t}-RA{W}_{i}}{TA{W}_{i}-RA{W}_{i}} & \,if\,RA{W}_{i}\le {S}_{i,t-1}\le TA{W}_{i}\\ 0 & \,if\,{S}_{i,t-1} < RA{W}_{i}\end{array}$$

where *F*_*max*_ $\left(\frac{mm}{day}\right)$ is the maximum infiltration rate depending on soil type^24^. In time steps where the sum of balance (i.e., *S*_*i,t-1*_ + *P*_*eff*_ - *Et*_*a,i,t*_ - *D*_*i,t*_) is negative, the *ET*_*a,i,t*_ and *D*_*i,t*_ were scaled proportionally in order to close the balance. In time steps where the sum of the balance (i.e., *S*_*i,t-1*_ + *P*_*eff*_ - *Et*_*a,i,t*_ - *D*_*i,t*_) is positive and exceeds *TAW*_*i*_, *R*_*i,t*_ – the sub-surface runoff – is calculated as the difference between the sum of the balance and *TAW*_*i*_.

For each day, each crop, and each grid cell within a MIRCA2000 region for which data on growing period was available, we calculated a stress *ET*_*a,i,t,s*_ – equal to the ‘green’ crop water requirement – and unstressed *ET*_*a,i,t,u*_ – equal to the actual evapotranspiration under no water stress *ET*_*i,t,s*_. ‘Blue’ crop water requirement was calculated as the difference between *ET*_*a,i,t,s*_ and *ET*_*a,i,t,u*_ and was only considered for irrigated areas. We then took a summation of the daily ‘green’ and ‘blue’ crop water requirements across each month of a crop’s growing season to determine monthly ‘green’ (for rainfed and irrigated crops) and ‘blue’ (for irrigated crops only) consumptive crop water requirements (Table S1). These definitions of ‘green’ and ‘blue’ crop water requirements are consistent with standard methodologies of water footprint calculation^1,4^.

### Model initial and non-growing season conditions

The model was initialized assuming an initial soil moisture condition of 50% of *TAW*. Following Hoogeveen *et al*.^14^, the model was then run for three years prior to the study start date using three randomly selected years of climate data. Because we ran multiple simulations (one for each crop), these three randomly selected years were held constant across simulations. For the months that fell outside of the growing season, we assumed a *k*_*c*_ value of 0.5. We also examined the sensitivity of our results to this off-season k_c_ value and found only limited variation.

## Data Records

Each monthly time-step of the dataset has global coverage (180°E–180°W; 90°S–90°N) with a 5 arcminute resolution (~10 km at the equator) and is provided in a standard WGS84 coordinate system. The data are provided in NetCDF-4 format, where the third dimension represents the crop type and the month for yearly and monthly results respectively. For each of the 5 major crops, a separate NetCDF-4 file was created. Green crop water requirements represent the amount of *ET* (i.e., actual crop-specific evapotranspiration demand (mm)) met by precipitation. Blue water requirements represent the amount of irrigation required to make up the difference between *ET* and the green crop water requirement after accounting for vertical soil water balances. Tables 1–3 report the names of each file and the main characteristics. These data files are also accompanied by a readme text file.

**Table 1:** Yearly (2000 and 2016) global maps of green and blue water for the 23 crops and 3 crop groups for a total of 156 maps. Unit is mm.

File – Folder year 2000 and 2016	Coordinate system	Number of rows	Number of columns	Resolution	Unit
BW_irrig_YEAR	WGS84	2160	4320	0.083333	mm
GW_irrig_YEAR	WGS84	2160	4320	0.083333	mm
GW_rainf_YEAR	WGS84	2160	4320	0.083333	mm

**Table 2:** Monthly global maps of green and blue water for the 5 main crops for a total of 60 maps. Unit is mm.

File – Folder monthly map year 2000	Coordinate system	Number of rows	Number of columns	Resolution	Unit
BW_crop-name_irr_2000	WGS84	2160	4320	0.083333	mm
GW_crop-name_irr_2000	WGS84	2160	4320	0.083333	mm
GW_crop-name_rfc_2000	WGS84	2160	4320	0.083333	mm

**Table 3:** Crop code and crop name.

Code	Crop	Code	Crop	Code	Crop	Code	Crop
1	Wheat	8	Soybeans	15	Rapeseed	22	Cocoa
2	Maize	9	Sunflower	16	Groundnuts	23	Coffee
3	Rice	10	Potatoes	17	Pulses	24	Others perennial
4	Barley	11	Cassava	18	Citrus	25	Fodder grasses
5	Rye	12	Sugar cane	19	Date palm	26	Others annual
6	Millet	13	Sugar beets	20	Grapes		
7	Sorghum	14	Oil palm	21	Cotton		

Yearly and monthly crop specific blue and green water requirement data are available in the freely and publicly available repository in *figshare* (10.6084/m9.figshare.c.4893084*)*^25^.

## Technical Validation

Our model shows that 5414 km^3^ of green water and 1068 km^3^ of blue water were consumed for crop production in the year 2000. Of these volumes, 922 km^3^ of green water and 1068 km^3^ of blue water were consumed on irrigated land covering 25% of total global harvested area, and the remaining 4491 km^3^ of green water were used by rainfed area. These cumulative results were validated against those of Siebert and Döll^7^, showing good agreement (a discrepancy lower than 3%) in both rainfed and irrigated conditions. Crop-specific results are reported in Table 4. Except for rye and rapeseed, whose water requirements are low compared to other crops, we observed differences lower than 15% for both rainfed and irrigated conditions.

**Table 4:** Global annual results for crop for the year 2000 and comparison against Siebert and Döll^7^.

Crop list	Data		WATNEEDS results			Data from Siebert and Doll (2010)	
	Area [Mha]	Percentage irrigated	Total Green Water [km^3^]	Total Blue Water [km^3^]	Percentage green water in irrigated area	Green Water [km^3^]	Blue Water [km^3^]
Wheat	214.4	31%	692.6	197.1	19%	650	208
Maize	151.3	20%	627.2	76.3	15%	585	72
Rice	164.9	62%	636.3	272.8	53%	634	307
Barley	55.1	8%	155.1	10.2	6%	150	11
Rye	10.4	4%	39.2	1.0	3%	12	1
Millet	33.6	5%	118.3	4.0	4%	132	4
Sorghum	40.1	9%	164.2	10.7	6%	173	11
Soybeans	74.8	8%	380.9	14.0	6%	382	17
Sunflower	20.8	6%	77.0	3.9	5%	68	4
Potatoes	19.7	19%	61.5	12.7	14%	61	14
Cassava	23.1	0%	131.1	0.0	0%	<0,05	<0,05
Sugar cane	20.7	49%	162.2	65.1	42%	173	69
Sugar beets	6.2	25%	21.1	8.4	16%	20	9
Oil palm	9.6	0%	99.7	0.0	0%	117	0
Rapeseed	24.6	14%	56.8	6.7	8%	51	8
Groundnuts	22.7	16%	84.7	7.3	15%	90	8
Pulses	67.0	8%	170.6	21.9	4%	173	22
Citrus	7.4	47%	42.6	21.5	37%	46	23
Date palm	0.9	79%	2.0	10.6	70%	2	11
Grapes	7.1	24%	26.8	7.1	19%	25	7
Cotton	33.1	49%	127.0	85.4	35%	131	84
Cocoa	6.7	0%	60.4	0.0	0%	66	<0,05
Coffee	10.1	2%	95.8	1.0	1%	102	1
Others perennial	72.9	18%	475.5	81.3	11%	515	84
Fodder grasses	104.5	11%	567.0	91.8	8%	576	90
Others annual	108.3	18%	338.0	57.5	11%	344	62
Total	1310	20%	5414	1068	16%	5278	1126

Globally, the crops accounting for the largest volumes of water consumption are maize (12% and 26% of green and blue water respectively), wheat (13% and 18% of green and blue water respectively) and rice (12% and 7% of green and blue water respectively), which together cover more than 40% of the total global harvested area. Though cultivated less extensively, the crops with the highest blue water requirements were date palm (1174 mm), sugarcane (305 mm), and citrus (300 mm).

For maps centered on the year 2000, we also performed a crop-by-crop pixel-by-pixel comparison to the dataset of Siebert and Döll^7^ for 23 main crops and 3 crop groups. The difference between Siebert and Doll and our model results in term of CWR is lower than 20% for about the 90% of the harvested area. All comparisons for rainfed and irrigated crops for the year 2000 are provided in the Supplementary Materials. For irrigated rice and wheat, which can have more than one growing period in the same location, the comparison was done considering only the main harvested period (i.e., the period associated to the larger harvested area). For 23 out of 26 crops, values of rainfed crops estimated by our model differ less than 20% compared with rainfed results by Siebert and Döll^7^, while 13 and 17 out of 26 for green and blue water (respectively) in irrigated areas showed an overall good agreement for crop water requirement.

For 5 major crops – which account for almost half of total crop production – we generated results at a monthly time scale and compared with MODIS evapotranspiration estimates^26^. We observed better agreement for rainfed crops with a discrepancy lower than 20% in 69%, 51%, 46%, 41% and 27% respectively for rainfed rice, sugarcane, soybeans, maize and wheat and an average of 20% for irrigated crops. Generally, the results from our model show higher values, likely due to the fact that in practice the full amount of required water is not provided to the field in many cases (i.e., deficit irrigation).

**Fig. 1:**
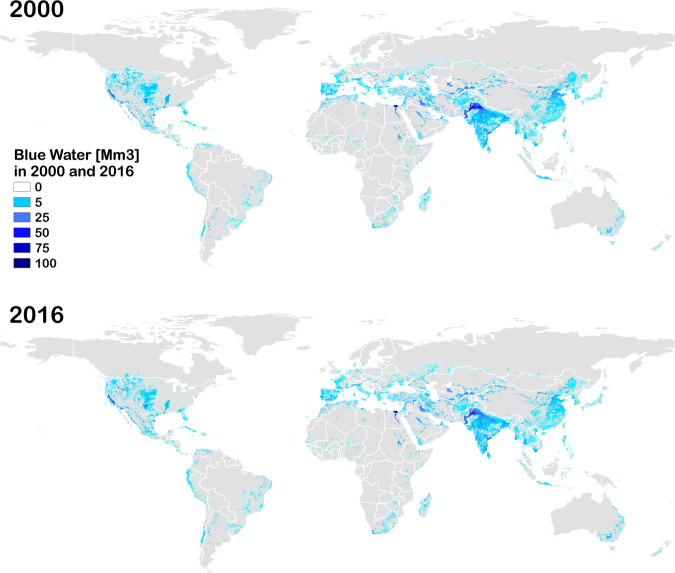
WATNEEDS results of blue water for total irrigated areas in 2000 and in 2016.

In the year 2016, crop production consumed 5740 km^3^ of green water and 1005 km^3^ of blue water, with an increase of 6% in green water consumption and a reduction of 6% of blue water relative to the year 2000 (Table 5 and Fig. 1).

**Table 5:** Green and blue water for the year 2016.

Crop year 2016	Total Green Water [km^3^]	Total Blue Water [km^3^]	Percentage of green water from irrigated land
Wheat	760	181	20.2%
Maize	682	65	16.0%
Rice	658	253	54.3%
Barley	164	10	6.0%
Rye	40	1	3.4%
Millet	127	3	4.2%
Sorghum	172	10	5.9%
Soybeans	421	11	6.5%
Sunflower	83	4	4.4%
Potatoes	65	13	13.7%
Cassava	133	0	0.0%
Sugar cane	168	63	41.9%
Sugar beets	22	9	15.5%
Oil palm	100	0	0.1%
Rapeseed	59	7	7.8%
Groundnuts	85	7	15.2%
Pulses	175	22	4.8%
Citrus	45	21	38.3%
Date palm	2	11	68.1%
Grapes	28	7	19.4%
Cotton	142	76	37.3%
Cocoa	60	0	0.2%
Coffee	96	1	1.3%
Others perennial	484	83	11.7%
Fodder grasses	616	91	8.0%
Others annual	354	57	10.9%
**Total**	**5741**	**1005**	**16.0%**

### Sensitivity analysis

We also performed sensitivity analyses on the initial condition and kc values during the non-growing season of the plant. Negligible changes (i.e., lower than 1%) are registered when changing the initial soil moisture condition from 0% to 100% as a result of the 3 year model spin-up to equilibrate the initial condition.

Varying kc values from 0.1 to 1 in the non-growing season produced a final variation in our estimates of less than 15% for green water and 20% for blue water (with the exceptions of rapeseed and rye) (Table 6). An average value of 0.5 was ultimately used, in order to include an average condition between bare soils, grassland, or second crops that could potentially cover the harvested areas in the non-growing season.

**Table 6:** Global crop water requirement with 3 different kc values during the non-growing season of the crop.

Crop	kc = 1.0		kc = 0.1		kc = 0.5	
	Green Water [km^3^]	Blue Water [km^3^]	Green Water [km^3^]	Blue Water [km^3^]	Green Water [km^3^]	Blue Water [km^3^]
Wheat	639	219	742	177	692.6	197.1
Maize	593	87	650	73	627.2	76.3
Rice	628	279	661	258	636.3	272.8
Barley	148	11	167	10	155.1	10.2
Rye	39	1	41	1	39.2	1.0
Millet	116	4	126	4	118.3	4.0
Sorghum	157	12	172	11	164.2	10.7
Soybeans	366	16	390	14	380.9	14.0
Sunflower	71	4	84	4	77.0	3.9
Potatoes	61	13	64	12	61.5	12.7
Cassava	132	0	135	0	131.1	0.0
Sugar cane	166	63	166	63	162.2	65.1
Sugar beets	20	9	22	8	21.1	8.4
Oil palm	99	0	99	0	99.7	0.0
Rapeseed	53	8	63	6	56.8	6.7
Groundnuts	83	8	90	7	84.7	7.3
Pulses	156	24	182	21	170.6	21.9
Citrus	42	22	42	22	42.6	21.5
Date palm	2	11	2	11	2.0	10.6
Grapes	26	7	26	7	26.8	7.1
Cotton	119	89	131	82	127.0	85.4
Cocoa	61	0	61	0	60.4	0.0
Coffee	96	1	96	1	95.8	1.0
Others perennial	476	82	476	82	475.5	81.3
Fodder grasses	569	93	569	93	567.0	91.8
Others annual	319	62	367	52	338.0	57.5
Total	5234	1127	5626	1019	5414	1068

## Usage Notes

The primary use of this dataset is to examine spatial and temporal trends of crop water use in tandem. Combined with estimates of cropland extent through time, this information now allows for historical estimates of water demand for global crop production and the incorporation of spatio-temporal variability and uncertainty into such assessments. With that said, there are several important issues about which users should be aware when utilizing this dataset:

- This dataset utilizes the primary planting and harvesting dates for each crop in each region. However, some regions have multiple cropping seasons^27^. It is therefore important that any region-specific analyses using this data incorporate a detailed understanding of that area’s cropping systems.
- Changes in atmospheric CO_2_ concentrations can produce competing effects on the water use efficiency of a crop. On one hand, elevated CO_2_ concentrations lead to increased air temperatures and greater plant transpiration. On the other hand, elevated CO_2_ concentrations permit smaller stomatal opening for carbon fixation. While these effects have been shown to largely cancel out for the time period 1981–2013 for rice, soybeans, wheat (C3 crops), and maize (a C4 crop), the water use efficiency of maize was found to be lower than estimated by a Penman-Monteith approach – as employed here – when considering longer time periods^28^. Thus while we expect that changing CO_2_ concentrations do not significantly affect our estimates of crop water requirements for C4 crops (e.g., maize, millet, sorghum, sugarcane), such considerations are essential for studies seeking to apply our methodology over longer time periods.
- Blue crop water requirements are not necessarily the same as the irrigation water that crops are able to receive. In some places with access to irrigation, supporting infrastructure may be insufficient to provide the water needed to avoid crop water stress^29^. To the extent possible, studies using this dataset (or any other global gridded dataset of estimating crop water requirements) should incorporate available information on actual irrigation water withdrawals and consumption to avoid overestimation.

This dataset provides an important temporal extension of existing global gridded estimates of crop water requirements and can also be useful in hydro-economic modelling and assessments of historical climate variability and trends in water scarcity.

## Supplementary information

Supplementary Information

## Data Availability

Code for calculating yearly and monthly crop specific blue and green water requirements is available in the freely and publicly available repository in *figshare (*10.6084/m9.figshare.c.4893084*)*^25^.
