# Supplementary material for: The green and blue crop water requirement WATNEEDS model and its global gridded outputs
Source: Sci Data. 2020 Aug 18;7:273. doi: 10.1038/s41597-020-00612-0 (PMC7434899; doi:10.1038/s41597-020-00612-0)
Supplement: Supplementary file 1 — Supplementary Information [file 41597_2020_612_MOESM1_ESM.docx]

**Supplementary Information**

Spatially distributed, temporal and crop specific comparison between WATNEEDS results and Siebert and Doll (2010) results for the year 1998-2002.

| **Year 1998** | IRRIGATED | | | | | | | | | | RAINFED | | | |
| --- | --- | --- | --- | --- | --- | --- | --- | --- | --- | --- | --- | --- | --- | --- |
| **Crop** | **Number of cells irrigated** | **BW**  **±20%** | **BW**  **>+20%** | **BW**  **< -20%** | **GW**  **±20%** | **GW**  **>20%** | **GW**  **< -20%** | **CWR**  **±20%** | **CWR >+20%** | **CWR**  **< -20%** | **Number of cells rainfed** | **GW**  **±20%** | **GW >+20%** | **GW**  **< -20%** |
| **wheat** | 6220 | 36% | 31% | 27% | 44% | 39% | 17% | 74% | 21% | 5% | 18260 | 42% | 50% | 8% |
| **maize** | 7742 | 34% | 21% | 40% | 60% | 25% | 15% | 92% | 3% | 6% | 23856 | 68% | 9% | 23% |
| **rice** | 6614 | 39% | 14% | 47% | 42% | 43% | 15% | 78% | 12% | 10% | 8114 | 57% | 12% | 32% |
| **barley** | 2599 | 28% | 36% | 29% | 52% | 20% | 28% | 87% | 7% | 6% | 12898 | 62% | 9% | 29% |
| **rye** | 390 | 26% | 33% | 28% | 58% | 22% | 19% | 88% | 6% | 6% | 5324 | 73% | 8% | 19% |
| **millet** | 1259 | 29% | 37% | 26% | 59% | 16% | 24% | 96% | 3% | 2% | 7610 | 59% | 4% | 37% |
| **sorghum** | 1461 | 34% | 25% | 33% | 64% | 16% | 21% | 96% | 1% | 3% | 9269 | 60% | 8% | 32% |
| **soybeans** | 2741 | 24% | 21% | 55% | 78% | 9% | 13% | 88% | 2% | 10% | 10307 | 75% | 4% | 21% |
| **sunflower** | 1159 | 37% | 23% | 39% | 64% | 19% | 17% | 93% | 3% | 4% | 7088 | 63% | 15% | 23% |
| **potatoes** | 3281 | 48% | 19% | 33% | 58% | 22% | 20% | 94% | 2% | 4% | 10506 | 68% | 11% | 21% |
| **cassava** | 14 | 71% | 29% | 0% | 29% | 57% | 14% | 79% | 21% | 0% | 6325 | 2% | 0% | 98% |
| **sugarcane** | 3456 | 46% | 27% | 24% | 62% | 9% | 28% | 92% | 1% | 7% | 3542 | 62% | 5% | 33% |
| **sugarbeets** | 920 | 58% | 14% | 26% | 52% | 33% | 15% | 91% | 5% | 4% | 3346 | 79% | 8% | 13% |
| **oil palm** | 9 | 89% | 11% | 0% | 44% | 0% | 56% | 100% | 0% | 0% | 2602 | 47% | 3% | 50% |
| **rapeseed** | 847 | 43% | 11% | 46% | 28% | 57% | 15% | 98% | 0% | 1% | 6583 | 66% | 14% | 21% |
| **groundnuts** | 2021 | 30% | 22% | 47% | 76% | 11% | 14% | 94% | 2% | 4% | 8304 | 54% | 3% | 43% |
| **pulses** | 2782 | 63% | 19% | 17% | 46% | 28% | 26% | 94% | 1% | 4% | 20491 | 62% | 20% | 18% |
| **citrus** | 2865 | 50% | 19% | 29% | 62% | 18% | 20% | 94% | 2% | 5% | 4108 | 85% | 5% | 10% |
| **date palm** | 381 | 71% | 12% | 17% | 34% | 41% | 25% | 94% | 3% | 3% | 242 | 32% | 32% | 36% |
| **grapes** | 921 | 48% | 18% | 33% | 54% | 29% | 16% | 90% | 6% | 4% | 3388 | 63% | 28% | 9% |
| **cotton** | 3388 | 43% | 25% | 31% | 59% | 20% | 21% | 93% | 3% | 4% | 5481 | 69% | 20% | 11% |
| **cocoa** | 7 | 29% | 14% | 57% | 71% | 0% | 29% | 100% | 0% | 0% | 2175 | 80% | 7% | 13% |
| **cofee** | 138 | 51% | 14% | 35% | 64% | 11% | 25% | 87% | 0% | 13% | 4295 | 79% | 9% | 13% |
| **others perennual** | 6890 | 52% | 21% | 26% | 68% | 12% | 20% | 93% | 2% | 5% | 18910 | 79% | 9% | 12% |
| **fodder grasses** | 5183 | 56% | 16% | 28% | 67% | 10% | 23% | 86% | 3% | 10% | 19931 | 73% | 11% | 16% |
| **others annual** | 6294 | 23% | 49% | 23% | 27% | 61% | 11% | 45% | 52% | 3% | 24183 | 50% | 40% | 10% |
| **Average** | **69582** | **44%** | **22%** | **31%** | **55%** | **24%** | **21%** | **89%** | **6%** | **5%** | **247138** | **62%** | **13%** | **25%** |
|  |  |  |  |  |  |  |  |  |  |  |  |  |  |  |
| **Year 1999** | IRRIGATED | | | | | | | | | | RAINFED | | | |
| **Crop** | **Number of cells irrigated** | **BW**  **±20%** | **BW**  **>+20%** | **BW**  **< -20%** | **GW**  **±20%** | **GW**  **>20%** | **GW**  **< -20%** | **CWR**  **±20%** | **CWR >+20%** | **CWR**  **< -20%** | **Number of cells rainfed** | **GW**  **±20%** | **GW >+20%** | **GW**  **< -20%** |
| **wheat** | 6290 | 38% | 26% | 32% | 37% | 51% | 12% | 75% | 21% | 4% | 18237 | 39% | 53% | 7% |
| **maize** | 7766 | 35% | 25% | 39% | 54% | 23% | 23% | 92% | 3% | 5% | 23960 | 65% | 10% | 25% |
| **rice** | 6492 | 38% | 14% | 49% | 38% | 42% | 19% | 81% | 9% | 10% | 7843 | 56% | 11% | 33% |
| **barley** | 2575 | 31% | 42% | 22% | 40% | 19% | 41% | 88% | 6% | 6% | 12838 | 60% | 12% | 27% |
| **rye** | 371 | 35% | 32% | 24% | 48% | 29% | 23% | 89% | 6% | 5% | 5414 | 72% | 16% | 13% |
| **millet** | 1277 | 37% | 46% | 14% | 46% | 19% | 35% | 95% | 4% | 1% | 7730 | 56% | 7% | 37% |
| **sorghum** | 1473 | 38% | 34% | 26% | 51% | 20% | 28% | 96% | 2% | 2% | 9394 | 60% | 8% | 32% |
| **soybeans** | 2674 | 27% | 27% | 45% | 66% | 9% | 25% | 90% | 2% | 8% | 10219 | 66% | 5% | 29% |
| **sunflower** | 1100 | 38% | 27% | 32% | 59% | 17% | 25% | 93% | 3% | 4% | 7084 | 59% | 17% | 25% |
| **potatoes** | 3400 | 51% | 24% | 25% | 52% | 18% | 30% | 93% | 2% | 5% | 10576 | 61% | 11% | 28% |
| **cassava** | 3 | 33% | 33% | 33% | 33% | 33% | 33% | 33% | 67% | 0% | 6252 | 1% | 0% | 99% |
| **sugarcane** | 3500 | 46% | 29% | 20% | 62% | 7% | 32% | 91% | 2% | 7% | 3657 | 63% | 5% | 33% |
| **sugarbeets** | 991 | 55% | 18% | 25% | 46% | 32% | 22% | 91% | 5% | 4% | 3464 | 65% | 13% | 22% |
| **oil palm** | 4 | 25% | 75% | 0% | 25% | 0% | 75% | 100% | 0% | 0% | 2666 | 56% | 1% | 42% |
| **rapeseed** | 808 | 47% | 15% | 37% | 25% | 58% | 18% | 98% | 0% | 2% | 6512 | 60% | 20% | 20% |
| **groundnuts** | 1982 | 30% | 32% | 37% | 65% | 10% | 25% | 93% | 1% | 5% | 8349 | 56% | 4% | 40% |
| **pulses** | 2717 | 66% | 18% | 15% | 38% | 34% | 28% | 94% | 2% | 4% | 20079 | 57% | 24% | 19% |
| **citrus** | 2885 | 52% | 19% | 27% | 64% | 18% | 17% | 94% | 2% | 4% | 4153 | 84% | 8% | 9% |
| **date palm** | 391 | 75% | 14% | 11% | 36% | 33% | 31% | 95% | 4% | 0% | 246 | 20% | 41% | 39% |
| **grapes** | 887 | 52% | 23% | 26% | 57% | 25% | 18% | 92% | 5% | 3% | 3351 | 62% | 29% | 8% |
| **cotton** | 3304 | 42% | 27% | 29% | 54% | 21% | 25% | 93% | 4% | 3% | 5358 | 70% | 20% | 10% |
| **cocoa** | 2 | 0% | 100% | 0% | 50% | 0% | 50% | 100% | 0% | 0% | 2087 | 78% | 6% | 16% |
| **cofee** | 157 | 46% | 22% | 31% | 54% | 15% | 31% | 85% | 3% | 12% | 4572 | 79% | 8% | 13% |
| **others perennual** | 6797 | 53% | 22% | 25% | 66% | 15% | 19% | 90% | 3% | 7% | 19085 | 76% | 11% | 12% |
| **fodder grasses** | 5251 | 55% | 15% | 30% | 65% | 15% | 20% | 89% | 3% | 8% | 19723 | 71% | 16% | 14% |
| **others annual** | 6483 | 25% | 52% | 20% | 26% | 62% | 11% | 45% | 52% | 3% | 24093 | 48% | 40% | 12% |
| **Average** | **69580** | **41%** | **31%** | **26%** | **48%** | **24%** | **28%** | **88%** | **8%** | **4%** | **246942** | **59%** | **15%** | **25%** |
|  |  |  |  |  |  |  |  |  |  |  |  |  |  |  |
| **Year 2000** | IRRIGATED | | | | | | | | | | RAINFED | | | |
| **Crop** | **Number of cells irrigated** | **BW**  **±20%** | **BW**  **>+20%** | **BW**  **< -20%** | **GW**  **±20%** | **GW**  **>20%** | **GW**  **< -20%** | **CWR**  **±20%** | **CWR >+20%** | **CWR**  **< -20%** | **Number of cells rainfed** | **GW**  **±20%** | **GW >+20%** | **GW**  **< -20%** |
| **wheat** | 6251 | 36% | 30% | 30% | 38% | 52% | 10% | 72% | 23% | 5% | 18034 | 40% | 54% | 6% |
| **maize** | 7681 | 33% | 23% | 41% | 56% | 24% | 20% | 90% | 3% | 7% | 24066 | 67% | 9% | 24% |
| **rice** | 6621 | 37% | 42% | 21% | 32% | 59% | 9% | 52% | 44% | 4% | 8031 | 53% | 12% | 35% |
| **barley** | 2548 | 36% | 36% | 22% | 45% | 19% | 35% | 86% | 9% | 5% | 12937 | 64% | 11% | 25% |
| **rye** | 384 | 33% | 32% | 21% | 59% | 21% | 19% | 89% | 9% | 2% | 5156 | 73% | 12% | 14% |
| **millet** | 1204 | 38% | 31% | 21% | 52% | 22% | 26% | 94% | 6% | 1% | 7653 | 55% | 6% | 39% |
| **sorghum** | 1510 | 39% | 30% | 24% | 50% | 21% | 29% | 94% | 4% | 2% | 9225 | 59% | 6% | 35% |
| **soybeans** | 2780 | 31% | 23% | 46% | 74% | 7% | 20% | 87% | 2% | 11% | 10352 | 74% | 4% | 22% |
| **sunflower** | 1147 | 45% | 22% | 33% | 60% | 21% | 18% | 94% | 3% | 3% | 7167 | 57% | 19% | 24% |
| **potatoes** | 3367 | 51% | 23% | 25% | 56% | 19% | 25% | 93% | 2% | 5% | 10682 | 65% | 12% | 22% |
| **cassava** | 12 | 67% | 17% | 17% | 17% | 83% | 0% | 67% | 25% | 8% | 6274 | 2% | 0% | 98% |
| **sugarcane** | 3612 | 45% | 26% | 24% | 63% | 10% | 27% | 92% | 2% | 6% | 3562 | 69% | 5% | 25% |
| **sugarbeets** | 945 | 57% | 16% | 27% | 48% | 35% | 17% | 94% | 4% | 3% | 3514 | 65% | 13% | 21% |
| **oil palm** | 8 | 100% | 0% | 0% | 38% | 0% | 63% | 100% | 0% | 0% | 2647 | 55% | 2% | 44% |
| **rapeseed** | 825 | 64% | 5% | 31% | 26% | 60% | 14% | 97% | 0% | 2% | 6491 | 59% | 24% | 18% |
| **groundnuts** | 1977 | 40% | 29% | 31% | 75% | 8% | 17% | 91% | 2% | 6% | 8478 | 56% | 4% | 41% |
| **pulses** | 2752 | 71% | 14% | 15% | 41% | 36% | 23% | 95% | 2% | 3% | 20161 | 59% | 26% | 15% |
| **citrus** | 2922 | 51% | 21% | 27% | 66% | 20% | 14% | 93% | 2% | 5% | 4084 | 83% | 8% | 9% |
| **date palm** | 381 | 71% | 13% | 16% | 25% | 53% | 22% | 91% | 8% | 2% | 280 | 39% | 39% | 23% |
| **grapes** | 922 | 53% | 22% | 24% | 59% | 30% | 11% | 93% | 6% | 2% | 3350 | 61% | 33% | 6% |
| **cotton** | 3343 | 48% | 23% | 28% | 53% | 26% | 21% | 92% | 3% | 5% | 5468 | 65% | 25% | 10% |
| **cocoa** | 4 | 25% | 0% | 75% | 100% | 0% | 0% | 100% | 0% | 0% | 2151 | 80% | 8% | 12% |
| **cofee** | 153 | 57% | 10% | 33% | 77% | 6% | 17% | 84% | 2% | 14% | 4490 | 79% | 11% | 10% |
| **others perennual** | 6772 | 54% | 21% | 25% | 68% | 19% | 12% | 93% | 2% | 4% | 18944 | 78% | 12% | 10% |
| **fodder grasses** | 5184 | 50% | 14% | 36% | 69% | 15% | 16% | 89% | 4% | 7% | 19711 | 72% | 17% | 12% |
| **others annual** | 6310 | 25% | 45% | 28% | 25% | 65% | 10% | 46% | 51% | 3% | 24050 | 51% | 39% | 10% |
| **Average** | **69615** | **48%** | **22%** | **28%** | **53%** | **28%** | **19%** | **87%** | **8%** | **4%** | **246958** | **61%** | **16%** | **24%** |
|  |  |  |  |  |  |  |  |  |  |  |  |  |  |  |
| **Year 2001** | IRRIGATED | | | | | | | | | | RAINFED | | | |
| **Crop** | **Number of cells irrigated** | **BW**  **±20%** | **BW**  **>+20%** | **BW**  **< -20%** | **GW**  **±20%** | **GW**  **>20%** | **GW**  **< -20%** | **CWR**  **±20%** | **CWR >+20%** | **CWR**  **< -20%** | **Number of cells rainfed** | **GW**  **±20%** | **GW >+20%** | **GW**  **< -20%** |
| **wheat** | 6396 | 37% | 26% | 33% | 38% | 51% | 10% | 74% | 22% | 4% | 18250 | 39% | 55% | 6% |
| **maize** | 7597 | 35% | 20% | 43% | 56% | 24% | 20% | 91% | 3% | 7% | 23699 | 66% | 10% | 25% |
| **rice** | 6378 | 41% | 17% | 42% | 39% | 44% | 17% | 78% | 12% | 10% | 7900 | 54% | 12% | 34% |
| **barley** | 2639 | 35% | 31% | 29% | 46% | 25% | 29% | 87% | 8% | 5% | 12820 | 63% | 13% | 25% |
| **rye** | 394 | 35% | 20% | 32% | 60% | 18% | 22% | 87% | 5% | 8% | 5335 | 75% | 11% | 14% |
| **millet** | 1280 | 43% | 24% | 27% | 53% | 24% | 23% | 96% | 3% | 1% | 7753 | 58% | 7% | 36% |
| **sorghum** | 1489 | 38% | 21% | 35% | 52% | 26% | 23% | 95% | 2% | 3% | 9323 | 59% | 9% | 33% |
| **soybeans** | 2626 | 33% | 24% | 42% | 66% | 11% | 23% | 85% | 2% | 12% | 10207 | 68% | 6% | 26% |
| **sunflower** | 1185 | 44% | 23% | 33% | 57% | 24% | 19% | 93% | 2% | 4% | 7069 | 62% | 16% | 22% |
| **potatoes** | 3345 | 52% | 23% | 25% | 52% | 21% | 27% | 93% | 3% | 4% | 10532 | 66% | 12% | 22% |
| **cassava** | 3 | 33% | 67% | 0% | 0% | 33% | 67% | 67% | 33% | 0% | 6282 | 2% | 0% | 97% |
| **sugarcane** | 3432 | 46% | 27% | 24% | 60% | 12% | 28% | 91% | 2% | 7% | 3628 | 65% | 6% | 30% |
| **sugarbeets** | 966 | 62% | 16% | 21% | 50% | 29% | 21% | 93% | 4% | 3% | 3417 | 66% | 12% | 22% |
| **oil palm** | 9 | 56% | 33% | 11% | 44% | 0% | 56% | 100% | 0% | 0% | 2706 | 47% | 2% | 52% |
| **rapeseed** | 835 | 68% | 6% | 27% | 23% | 66% | 11% | 99% | 0% | 1% | 6521 | 63% | 20% | 18% |
| **groundnuts** | 1964 | 37% | 31% | 32% | 65% | 11% | 24% | 92% | 2% | 6% | 8368 | 52% | 3% | 44% |
| **pulses** | 2607 | 70% | 14% | 15% | 40% | 35% | 25% | 94% | 2% | 4% | 20310 | 57% | 26% | 16% |
| **citrus** | 2900 | 54% | 16% | 29% | 62% | 24% | 14% | 94% | 2% | 4% | 4077 | 82% | 11% | 7% |
| **date palm** | 335 | 77% | 8% | 15% | 29% | 53% | 19% | 88% | 10% | 2% | 265 | 35% | 31% | 34% |
| **grapes** | 955 | 50% | 18% | 32% | 49% | 38% | 13% | 93% | 5% | 1% | 3291 | 56% | 36% | 7% |
| **cotton** | 3486 | 50% | 22% | 26% | 56% | 24% | 20% | 91% | 3% | 6% | 5443 | 65% | 27% | 8% |
| **cocoa** | 5 | 60% | 20% | 20% | 60% | 0% | 40% | 80% | 0% | 20% | 2108 | 77% | 10% | 13% |
| **cofee** | 130 | 41% | 25% | 35% | 53% | 24% | 23% | 88% | 0% | 12% | 4422 | 77% | 11% | 12% |
| **others perennual** | 6889 | 54% | 20% | 26% | 67% | 19% | 15% | 93% | 3% | 4% | 18908 | 76% | 13% | 11% |
| **fodder grasses** | 5175 | 52% | 15% | 34% | 69% | 16% | 15% | 90% | 4% | 6% | 19976 | 72% | 16% | 11% |
| **others annual** | 6297 | 26% | 46% | 25% | 24% | 66% | 10% | 45% | 52% | 3% | 24241 | 50% | 40% | 10% |
| **Average** | **69317** | **47%** | **24%** | **27%** | **49%** | **28%** | **24%** | **88%** | **7%** | **5%** | **246851** | **60%** | **16%** | **24%** |
|  |  |  |  |  |  |  |  |  |  |  |  |  |  |  |
| **Year 2002** | IRRIGATED | | | | | | | | | | RAINFED | | | |
| **Crop** | **Number of cells irrigated** | **BW**  **±20%** | **BW**  **>+20%** | **BW**  **< -20%** | **GW**  **±20%** | **GW**  **>20%** | **GW**  **< -20%** | **CWR**  **±20%** | **CWR >+20%** | **CWR**  **< -20%** | **Number of cells rainfed** | **GW**  **±20%** | **GW >+20%** | **GW**  **< -20%** |
| **wheat** | 6406 | 40% | 27% | 28% | 42% | 46% | 13% | 76% | 20% | 4% | 18204 | 39% | 53% | 8% |
| **maize** | 7617 | 39% | 24% | 34% | 59% | 19% | 21% | 93% | 2% | 5% | 23953 | 63% | 9% | 28% |
| **rice** | 6635 | 41% | 14% | 45% | 37% | 45% | 18% | 79% | 10% | 10% | 8038 | 53% | 11% | 35% |
| **barley** | 2605 | 37% | 35% | 21% | 48% | 20% | 33% | 89% | 6% | 4% | 12944 | 58% | 11% | 32% |
| **rye** | 416 | 36% | 24% | 24% | 53% | 29% | 18% | 93% | 5% | 2% | 5168 | 67% | 14% | 19% |
| **millet** | 1218 | 41% | 27% | 22% | 49% | 21% | 30% | 96% | 2% | 2% | 7731 | 50% | 6% | 44% |
| **sorghum** | 1494 | 42% | 24% | 24% | 55% | 20% | 25% | 96% | 2% | 2% | 9258 | 54% | 8% | 38% |
| **soybeans** | 2736 | 29% | 24% | 46% | 72% | 8% | 20% | 90% | 2% | 9% | 10267 | 64% | 5% | 32% |
| **sunflower** | 1112 | 37% | 24% | 38% | 57% | 22% | 21% | 95% | 2% | 4% | 7177 | 54% | 17% | 28% |
| **potatoes** | 3330 | 49% | 24% | 27% | 53% | 22% | 25% | 93% | 2% | 5% | 10370 | 60% | 11% | 29% |
| **cassava** | 12 | 33% | 25% | 42% | 58% | 33% | 8% | 83% | 17% | 0% | 6379 | 2% | 1% | 97% |
| **sugarcane** | 3519 | 48% | 29% | 21% | 60% | 9% | 31% | 91% | 1% | 7% | 3682 | 63% | 5% | 32% |
| **sugarbeets** | 901 | 49% | 27% | 23% | 48% | 34% | 18% | 92% | 5% | 2% | 3391 | 65% | 9% | 26% |
| **oil palm** | 11 | 91% | 9% | 0% | 91% | 0% | 9% | 100% | 0% | 0% | 2651 | 41% | 2% | 58% |
| **rapeseed** | 834 | 53% | 13% | 34% | 23% | 56% | 21% | 98% | 0% | 1% | 6648 | 59% | 17% | 24% |
| **groundnuts** | 1972 | 37% | 26% | 36% | 68% | 11% | 21% | 92% | 2% | 5% | 8354 | 43% | 2% | 55% |
| **pulses** | 2726 | 65% | 22% | 13% | 37% | 28% | 35% | 95% | 2% | 3% | 20201 | 58% | 20% | 22% |
| **citrus** | 2864 | 45% | 24% | 28% | 59% | 18% | 23% | 94% | 2% | 4% | 4067 | 80% | 6% | 14% |
| **date palm** | 408 | 75% | 6% | 19% | 37% | 45% | 18% | 96% | 1% | 2% | 254 | 25% | 44% | 31% |
| **grapes** | 917 | 50% | 22% | 28% | 56% | 28% | 16% | 92% | 6% | 2% | 3378 | 64% | 26% | 9% |
| **cotton** | 3322 | 45% | 26% | 28% | 53% | 23% | 24% | 92% | 3% | 5% | 5386 | 66% | 20% | 14% |
| **cocoa** | 6 | 50% | 17% | 33% | 83% | 0% | 17% | 83% | 0% | 17% | 2133 | 68% | 9% | 23% |
| **cofee** | 138 | 52% | 10% | 38% | 78% | 9% | 14% | 93% | 0% | 7% | 4347 | 76% | 9% | 15% |
| **others perennual** | 6784 | 48% | 26% | 25% | 64% | 16% | 19% | 94% | 3% | 4% | 18749 | 75% | 10% | 15% |
| **fodder grasses** | 5193 | 55% | 18% | 28% | 70% | 15% | 15% | 91% | 4% | 4% | 19612 | 73% | 14% | 12% |
| **others annual** | 6327 | 23% | 53% | 22% | 25% | 63% | 12% | 44% | 53% | 3% | 24086 | 49% | 40% | 11% |
| **Average** | **69503** | **47%** | **23%** | **28%** | **55%** | **25%** | **20%** | **90%** | **6%** | **4%** | **246428** | **56%** | **15%** | **29%** |
